# On Morbid Impulse
*The basis of this paper formed the *Browne* Prize Essay for 1860, Class of Med. Psychology and Mental Diseases, University of Edinburgh.


**Published:** 1863-01

**Authors:** W. Carmichael McIntosh

**Affiliations:** Murray's Royal Asylum, Perth.


### Art. VIII.—ON MORBID IMPULSE.*

By W. Carmichael McIntosh, M.D., Murray's Royal Asylum, Perth.

It is but a little while ago that the doctrine of impulsive mania, apart from other evident mental lesion, was countenanced by our legal authorities; and there is no doubt that many an unfortunate individual, worthy of a milder fate, satisfied apparent justice by death, or was consigned to languish his dreary existence in a gloomy prison. Now, however, from the progress of medical science, this form of mental derangement is recognised more clearly, and, as consequences, irresponsible beings are often saved from misdirected punishment, and society, and the afflicted themselves, from their insane tendencies. But even still there is scarcely a serious case of the kind which excites attention in our law courts without entailing much annoyance, anxiety, and perplexity to both legal and medical authorities; especially the latter, who, it cannot be doubted, are most fitted to judge, from their previous training and experience. As the great aim of the ordinary physician and surgeon is, either to prevent disease altogether, or else to treat it so early as to mitigate its asperities and check further advancement; so the alienist is expected now, with delicate sagacity, to be alive to all those intricate symptoms which indicate or precede cerebral aberration, as well as to treat the disease in its well-marked or chronic forms. So zealous, indeed, have some alienist writers become on the former point, that they have fallen into the grievous error of confounding crime with morbid impulse, thereby aggravating the doubt and perplexity, where both already existed too largely.

In the following essay I propose to examine (1) the Nature and (2) the Causes of Morbid Impulse.

---

* The basis of this paper formed the *Browne* Prize Essay for 1860, Class of Med. Psychology and Mental Diseases, University of Edinburgh.

1. *Nature.*—By morbid impulse, as a form of insanity, is meant that condition of the mental organism in which the individual is irresistibly or uncontrollaby impelled to commit some act, either unconsciously, or if conscious, without the power on his part to prevent it. It may occur in various states. The intellect may be congenitally defective, as in idiocy and imbecility; or the brain may not be much disordered, and the agent may be perfectly conscious that he is committing a deed which is culpable; or, again, the individual under the impulse does an act of the nature of which he afterwards loses all remembrance—a happy Lethe, perchance, for the dire result. This is sometimes seen in epilepsy, either before or after the fit. Lastly, the impulse may take place as one of the traits of character in the true lunatic, requiring constant and efficient surveillance to prevent serious injury to himself or others. In many cases the intellect, to all appearance, is little deranged, while, lurking in the secret recesses of the brain, there lies some sweeping moral defect or perversion, which only requires its peculiar stimulus to be excited into active mischief. Ordinary friends around do not dream of mental derangement, and even one examination by a physician may not always lead the patient to betray his defect, unless very skilfully handled; yet, in no long time, a desperate or disgraceful act may startle all—alike by its impetuous suddenness and dangerous nature. For instance, it is related that a gentleman of high attainments and character, while in the apparent enjoyment of excellent health and spirits, had a dinner party of his friends; there was no one present so agreeable and attractive in conversation and manners as himself; but in the middle of the festivity he rose and politely apologised for absenting himself a moment, and, retiring to an adjoining room, cut his throat to the vertebræ, at the very time when his friends were drinking his health.

Certain impulsive acts, compared with ordinary ones, are to the latter what the two kinds of muscular action are to each other; the one is voluntary, "since as the mind commands and wills, it may be excited, increased, diminished, or arrested; the other involuntary, of which the mind is either unconscious, or if conscious, the motion is performed without its consent, and is excited only by a corporeal stimulus applied to the nervous system. The one depends on the free will of the creature, the other is purely automatic and independent of it." Purely instinctive acts, however, are very different from examples of morbid impulse. Both excite a special class of conceptions, which constitute the object and satisfaction of the instinct, but in the former case, the instinct or impulse is natural, whereas in the latter it is unnatural or diseased. The latter (morbid impulse or instinct) is, however, almost invariably a perversion of the natural instinct, and hence the relation between the two. "A strong and wholly sensational desire, which arises from obscure sensational stimuli, and the material ideas of which are consequently little developed in the brain, is termed *a blind impulse* (*instinct, sympathy, sensual propensity, sensual inclination, natural instinct generally*)." These blind instincts or impulses "are divided into the instincts of *self-preservation, self-maintenance*, the *propagation of the species*, and *love of offspring*."* It will at once be seen that the perversions (from disease) of these last-mentioned instincts constitute the characteristic and leading varieties of morbid impulse; for instance, Nature has endowed all animals with an instinctive love of life, and an abhorrence of the opposite. We see that timid animals fly from the object of their dread or hide themselves in secure places; and if the stronger brutes wage fierce combat with the invader, it is not because they are careless of life, but rather that, by exerting themselves to conquer, they strive to retain that which is in jeopardy. Man in a state of nature seldom or never commits suicide, because his instincts and impulses are natural, even though they may be exaggerated, whereas in civilization nothing is more common, because our natural instincts are prone, by a multitude of circumstances, hereafter to be noticed, to disease and perversion.

In morbid impulse, then, certain of the moral faculties and instincts seem to be congenitally defective (and this form manifests itself in early life) or are destroyed or perverted by disease, inaction, or injury. If we regard the cerebrum as the overruling power, and the cerebellum and cerebral ganglia the means by which in ordinary circumstances volition is exercised, and the various external relations acquired, then it would seem that in these cases part of the connecting nerve substance, by which the cerebrum governs and directs by moral sway our various acts, was diseased or otherwise rendered incapable, while the act itself was carried out with vigorous energy. The other faculties remaining in tolerable condition, there does not necessarily follow an unconsciousness of the impulsive act; indeed the person may be fully aware of it, and yet by the defect before mentioned be incapable of staying it. The laws of unconscious central action, as developed by Professor Laycock, have an important bearing on this question, for just as certain cries are automatic, so may acts. "The cerebral and cerebellar hemispheres (he writes) may be considered as extensive peripheries, having, like the corporeal periphery, the medulla oblongata for their centre. So that tele-

* Unzer's *Principles of Physiology*, translated and edited by Prof. Laycock, p. 57.

organic changes taking place therein, which in the usual states coincide with conscious states, as ideas, feelings, or desires, may during morbid states pass downwards to the medulla oblongata, and there excite the activity of appropriate motor or kinetic substrata, without at the same time exciting any state of consciousness whatever. This is, in fact, what occurs in all states of automatic cerebral action." "Possibly it is in the locus niger we must look for the common sensory."

Corporeal appetites are produced without consciousness, as is well known. An angry man can more readily assuage his passion without avenging himself than a hungry man can quiet his stomach without taking food. Nature impels animals to the blind use of the sexual organs with which she has supplied them, as well as with the knowledge how to use them; without their being aware that they will thereby propagate their species. It would appear, indeed, that no animal, except reasoning man, is aware of this subject. If, however, there be disease or degeneration of the nervous centre in connexion with the foregoing, then the person indulges madly in all brutal appetites and instincts. This is seen in a certain degree in that great and instructive class, our criminal population. Amongst these there is a great deficiency of that controlling power which by reason tempers our natural impulses, and keeps them within decorous limits. In such cases we wonder little at the committal of desperate acts; it is when a person of good character and attainments is hurried away by an irresistible inclination, impelled by a blind, *unnatural* instinct, that cannot be accounted for, to this or the other action which his reason reprobates and condemns—it is this which strikes us with astonishment. "Beset by ideas of robbery, of incendiarism, of murder, of suicide, which he in vain strives to combat, he feels all the horror of such desires, and yet his will is overborne; without a motive, without an interest, he burns, slays, or sheds his own blood."*

In that period of imperfect cerebral action which occurs between sleeping and waking, many striking examples of morbid impulse have been recorded. Among others is related the case of a pedlar who was sleeping by a roadside, when a countryman passing by shook him by the shoulders in joke; the man suddenly awoke, drew a sword-stick, and stabbed the disturber through the heart. This clearly was an irresponsible act, but the court did not think so, for the pedlar was hanged. This peculiar state will be more fully noted under the Causes.

Instances of morbid impulse, in one or other of its forms, have

---

* Dr. W. A. F. Browne, Reports of Dumfries Asylum.

† "Deviation maladire d'un type primité."—*Morel.*

been recorded from very early ages: the case of Cleomenes, king of Sparta, mentioned by Herodotus as being prone to violent assaults and other insane impulses, shows several interesting features. He was at length confined, and managing to circumvent his attendant and obtain a knife, he killed himself by making long gashes in his flesh. In the present day the affection is by no means uncommon. One of the most frequent varieties is the derangement of the sexual instinct. The victims of the latter, however, preserve silence as to their thraldom, until corporeal and mental vigour have yielded before the insane tendency. It is not rare for a mother, in whom not the slightest mental infirmity—usually so called—can be detected, to have enduring and deadly hatred against some one of her offspring, and on whom she wreaks such acts of violence and cruelty as to necessitate the removal of the child. The insane impulse for alcoholic stimuli is not uncommon; cases of suicide, homicide, and perhaps kleptomania, abound; and the same fatality leads many men to indulge to excess in opium, cannabis indica, and chloroform. Glancing at the morbid impulses of the veritable insane in our asylums, it is found that there are marked, if not the chief, features in the case of many patients. In almost every large asylum there are extreme cases of pica; patients who devour earth, filth, grass, &c.; many have enormous appetites, and besides finishing their own ample allowance, they eagerly devour all eatables they can lay their hands on, even to a quantity sufficient for four or five ordinary men. Cases of erotic impulse, satyriasis, suicide, homicide, and kleptomania are shown in vivid colours, and are reflected sharply from the oft enfeebled state of the intellect and will.

This impulsive insanity is limited to no age, and has been observed fully developed at a very early period of life. Throughout the pages of our literature on insanity are numerous cases where children of tender years have hanged or drowned themselves on the slightest grounds. Precocity of development in the generative organs, occurring generally at either dentition (three or seven years), leads to masturbation. The case of a little girl is recorded, who combined insane cunning with the erotic and suicidal impulses. The period of puberty is a fertile field for many examples; while adult age and the decline of life are each subject to inroads. The person affected may have an irresistible impulse to perform some trifling act, or he may blindly rush into homicide or suicide.

Sometimes the morbid impulse seems analogous to that class of animal instincts and passions which are aroused by the sight of bright colours, as scarlet to the bull and white to the bat. This is illustrated in the case of a girl, of unexceptionable morals and character, of mild and amiable deportment, who was attacked by the most intense impulse to kill a child when she was undress- it, apparently at the sight of its white skin. She beseeched her mistress to dismiss her, lest she should no longer be able to control the dreadful tendency so foreign to her feelings. In another group of cases, the excitant to the impulse appears to be the sight of a ready means of accomplishing it. Thus many many lofty towers required to be shut up from the public, so great was the number of unaccountable suicides therefrom. It is related, in regard to Strasburg cathedral,* "that three females have been, at different times, so overpowered by the giddy eminence which they had reached, that they have thrown themselves off in a momentary fit of delirium, and been dashed to atoms." It is in such instances as the foregoing that the greater affectability of the whole nervous fabric of woman renders her more liable to disease. Again, cases occur clearly testifying the seat of the disease to be in the mental faculties, and where it has been cured instantaneously by moral means alone. The well known case, related by Dr. Gregory, of a man who, while going to commit suicide by drowning, was attacked by a robber, &c., and who changed his thoughts by this means, and went home contented.

The tendency to the manifestation of impulsive insanity is often hereditary, just as other peculiar mental qualities and bodily states are. It is no uncommon thing to see the children of an impulsive suicide manifest the morbid condition in various other forms, such as excessive and brutalising sensual indulgence, dexterous and incorrigible thieving—particularly in the females, while other members may be rendered imbecile by alcohol.

An experienced writer† states that there are four ways in which the onset of the malady may arise:—"It may arise suddenly, strongly, irresistibly, and precipitate the actor into a course diametrically opposed to his previous character." This is well illustrated in the case of the gentleman committing suicide, previously related; or where a virtuous and chaste female becomes all at once vicious, depraved, bold in language and gesture, and utterly shameless. In such a case as the latter, most authors agree that these vicious and depraved tendencies, thus suddenly appearing, existed most probably in the mind of the person anteriorly, but had been heavily curbed by a dominant reason or the dictates of prudence. So soon as the restraining power becomes sapped by disease, the unbridled passions and instincts are cast loose. The case mentioned by

---

* Foot note to Dr. Winslow's *Obscure Diseases of the Brain, &c.*

† Dr. W. A. F. B:owne: *Reports, &c.*

Dr. Wigan of a young gentleman who had an ungovernable propensity to run up into an organ loft during divine service, and play some well known jocular tune, attached perhaps, to profane and indecent words. It was impossible to prevent him, so sudden was the act, and he voluntarily absented himself from chapel. "The impulse, again, may be the conclusion or completion of a series of irregularities. The patient may have been observed to conduct himself strangely, changing articles requiring no change, becoming excessively obstinate and irritable about trifles, indulging in unwonted potations or play, or otherwise not appearing to be the man he was before;" yet it may never strike any of his friends that brain disease is imminent, until some wild impulsive act explains all.

Another mode is that in which "a passion may be nursed and nourished until it obtain dominion over every other power." Thus, the device to steal, an exaggeration of our natural acquisitiveness, does not manifest itself when the governing power controls the tendency; but as the bonds of the moral sway grow slighter and slighter under the influence of disease, kleptomania is produced and developed, until at length the dominant idea of the patient is to steal all that can be lifted and to grasp what cannot. All the higher qualities must yield when this one reigns, and the individual steals and steals or thinks he steals, it may be unconsciously, and certainly without the power to prevent so culpable a tendency.

"A third form is that where tendencies in themselves hideous, long subdued by reason and religion, or disguised by prudence, are developed by the decay and deterioration of better principles, by external temptations." A sudden outbreak often reveals the existence of long suppressed vicious thoughts. It is no great rarity for patients to seek the gates of an asylum, when they feel the governing power waning, and entreat the physician to take care of them, since they can no longer take care of themselves; and well for such if no obstacle intervene. The misfortunes and vicissitudes of life are too much for the brains of many; rash acts of an impulsive nature betraying the lesion of the will. Again, a person removes to a new sphere of life, perhaps amongst loose companions and gay society—it may be too, alas! not of the best description; how easy is it for the thinned integument to give way, disclosing latent evils, and setting free terrible impulses, which till now had been kept in check.

Again, "the moral sense is warped as the will or the imagination is by cerebral disease." Many acts of eccentricity or insanity are thus to be accounted for in after life. The lives of many great men, however, who have reached an advanced age with their mental and moral powers undiminished, make it a gratuitous hypothesis to suppose that age necessarily brings with it degeneration and decay of these faculties.* Yet, as it is, we meet with a good many instances where lesion of the governing will is too evident. Some of the aged and irascible are something more. The gross sensuality of tottering old men, ensuing after a life of strict morality and uprightness, speaks sadly in favour of the latter, for in no other way can those extravagant vagaries be accounted for. The individual all his life long may have been struggling with these perverted instincts, and till now has conquered them; but either a sudden accession of brain disease saps reason's sway, or else the age-enfeebled force can no longer hold the citadel. An ably penned article by Barlow has it that "Man has in his own nature the antagonistic power, which, if properly used, can set at naught the evils—aye, and the so-called irresistible propensities too—of the bodily organism. So nicely balanced indeed is the machine, that a grain can turn it to either side, but it is in the power of the will to cast that grain: cast on the side of instinct, the propensity becomes passion, and the passion crime, and both are for the time insanity; for when once the will has lent its force to the blind impulses of the body, whether diseased or in health, it becomes only a question of time whether the individual is to be called insane and placed under restraint or not. The man who recovers quickly from his madness is called a sane man, though he may a few minutes before have exhibited the flushed face, rapid and violent gestures and language, and the unreasoning conclusions of a maniac; but, strange to say, if this be very frequent, he is excused and considered innocent of the crime he perpetrates, exactly because he has committed the greatest of all crimes by delivering over his god-like intellect to be the sport of the brute nature it ought to regulate."

In the lower animals, instincts are mainly uniform and permanent, with little actual intelligence in any case to modify or control them. For instance, the maternal instinct (of which infanticide is the unnatural or morbid form) is usually excited blindly. "The parent animal knows neither why she broods, nor what she hatches, or gives birth to. She tends, allures, covers, nourishes, and protects her young, blindly: nay, will perform these offices for young animals she has never known before, and which require attentions entirely different from those she affords, consequently without any knowledge or aims

* On this point *vide* Dr. Winslow *On Obscure Diseases of the Brain, &c.*, p. 681, *et seq.*

of the instinct." It is thus that hens will bring up ducklings, cats suckle rabbits and leverets, and linnets hatch and feed, to the destruction of their own offspring, the young of the cuckoo. Man's instincts, on the other hand, are fewer in number, and, though individual, have extensive relations with his moral nature, and are subject to his dominant intellect. By vigorous culture and the effects of an enlightened civilization, man strives to place these natural impulses or instincts in their legitimate positions, suppressing some, curbing others, and encouraging such as his intellect pronounces to be beneficial to society and himself. There is thus a marked difference between the lower animals and man ; for, in the former, however intelligent and wonderful many acts seem, still they are blindly or instinctively done ; whereas in the latter, reason tempers every instinct, and modifies it to the requirements of the being, with additional perception of the object and purpose of the act. It will readily be understood, then, that the temporary paralysis or disease of the organ of the intelligence, as evinced by the impairment of the all-powerful will, is the true source and explanation of morbid impulses.

2. *Causes.*—The causes of physical disease are as various as they are numerous in kind and degree ; and so it is with mental, the departures from the healthy tone of mind occurring in the slightest and most imperceptible, as well as in the intensest forms.

The causes of morbid impulses are many of them common to all forms of insanity, so that if, in the following detailed account of them, any seem far-fetched, due allowance must be made for this necessary extension of causation. Many cases, from their suddenness, are said to occur without the slightest premonitory symptoms, or appreciable cause ; but surely such a state of matters may, in most examples, be attributed rather to a want of a thorough and competent investigation of the previous character, habit, disposition, thoughts, constitution, and hereditary tendencies of the patient. We are not wont to affirm that any physical disease arises without cause, even although the special excitant or contagion may not be detected by the severest scrutiny; and it requires no stretch of fancy to predict that impulsive insanity cannot occur without exciting and predisposing causes, whether centric or eccentric.

It is well known that the modifications of all mental phenomena depend upon alteration in the functional activity of the brain ; a cessation of this activity, as in profound sleep, is coincident with an abolition of mental action. The cerebrum is made up of a series of ganglia, and it is with the dynamical changes which go on in these centres that mental phenomena are coincident. A manifest structural change in the entire organ can only happen coincidently with the abolition of the mental faculties, whereas in most forms of insanity the faculties remain, but are warped and perverted. When the functional changes which produce insanity have passed into structural alterations, and the intimate tissue is so compressed by congestion or effusion, or disintegrated —we have loss of mind, not disorder merely. If the action of the co-ordinating apparatus be interrupted, or a link of the chain removed in the intellect, feelings, emotions, appetite, and instincts, we have manifestations of disorder of the mind. It does not always follow, however, that this centric condition is the initiatory cause of the malady, more especially of that form termed morbid impulse. Unzer observes, that it is erroneous to say that the animal movements excited by the external impressions are mental, or, at least, cerebral. Hence have arisen those erroneous views, which have had so injurious influence on pathology and therapeutics, to the effect that all the phenomena of fevers, spasmodic diseases, epilepsy, paralysis, and all nervous diseases in general, depend upon some affection of the brain, and that they must be cured by remedies which act upon that viscus. On the contrary, an internal impression excited by nerves far distant from the brain by various irritating agents in the body, especially by reflected external impressions which are not felt, may induce all these affections, independently of the brain, and must be cured by the removal of these agents.

Occupying a prominent position alike as the cause of mental and physical disorder is *hereditary influence;* and no better field than the present could be afforded for the display of this all-powerful cause. Morbid impulse in one or other of its forms may be implanted in the mental conformation by hereditary predisposition. The accomplished physician sees it in the physiogonomy and external form, in the ideas, passions, habits, and inclinations of the offspring, and he may even predicate its occurrence. No better illustration of this occurs than in the morbid development of the love of life—that is to say, in persons remarkable for their egotism and fear of death or personal injury—a hyperæsthesia changed into a paræsthesia.* Several cases are recorded where whole families, sprung from suicidal parentage, have been suicidal, and blindly carried out their desperate impulse, it may be at one particular period of life. The following signal example from Gall will illustrate this hereditary influence clearly. The Sieur Gauthier, the owner of various houses built without the barriers of Paris, to be used as entrepôts of goods, left seven children, and a for-

* Prof. Laycock, *Mind and Brain,* vol. ii., p. 305.

tune of about 2,000,000 francs to be divided among them. All remained at Paris, or in the neighbourhood, and preserved their patrimony; some even increased it by commercial speculation. None of them met with any real misfortune, but all enjoyed good health, a competency, and general esteem. All, however, were possessed with a rage for suicide, and all seven succumbed to it within a space of thirty or forty years. Some hanged, some drowned themselves, and others blew out their brains. One of the first two had invited sixteen persons to dine with him on a Sunday. The company collected, the dinner was served, and the guests at the table. The master of the house was called, but did not answer; he was found hanging in the garret. Scarcely an hour before, he was quietly giving orders to the servants and chatting with his friends. The last, the owner of a house in the Rue de Richelieu, having raised his house two stories, became frightened at the expense, imagined himself ruined, and was anxious to kill himself. Thrice they prevented him; but soon after, he was found dead, having shot himself. The estate, after all debts were paid, amounted to 300,000 francs, and the owner might have been forty-five years old at the time of his death. The hereditary impulse for stimulants is often clearly defined; for instance, in a given case, one or other, or both, parents are drunken, how often are their children found treading in their footsteps! A well-marked case, within our own knowledge, is at present coursing to ruin; the son of a parent, who died at an early age from excessive intemperance; he possesses much resemblance to his father in form and features. From his earliest boyhood the fatal impulse was but too evident. It does not always follow that the hereditary taint of necessity influences one peculiar line of impulse; on the contrary, since the mental condition which favours one favours all or any, we may have the children of a dipsomaniac homicidal, suicidal, kleptomaniacal, or erotic. This peculiar mental constitution is clearly a gift of nature in many cases. In such a constitution the *vis nervosa* passes very rapidly, and all teleorganic changes are speedily incited.* A person of such a mental configuration in a hyperæsthetic state at once may commit irrèsistibly any act from a cause which would not have disturbed the equanimity of a more stolid neighbour. There is increased activity of the encephalon after a time, with loss of power of consciousness and the restraining faculties.

Further, though neither parent may manifest morbid impulse, yet the mental constitution of each may be such that the new being, the result of the fusion of the sperm and germ cells, may

* *Lectures on Med. Psychol. and Ment. Dis.*, Univer. Edin.

be a perverse and impulsive mortal. The mental state of the parents at the time of conception, too, requires consideration. "The sons of fathers rendered immortal by the power of their intellect and the sublimity of their genius, how often imbecile!" Men who have raised themselves to the pinnacle of fame by their untiring devotion to literary and scientific pursuits, have often descendants by no means worthy of such parentage. The state of the parents' mind at the time of conception, as above mentioned, would tend to throw some light on the solution of this difficulty: with a brain taxed to overstraining, and jaded without intermission—hyperæsthetic perhaps beyond a healthy limit, and with a frame worn by application, the parent has intercourse, the result of which, if conception takes place, must be a creature resembling one or other parent, or both. Who denies that mental states influence the mammary secretion? It may not be going too far to assert that the seminal fluid must be also subject to their influence in the male; and that, just as we have the mammary secretion, whose use is the nourishment of the infant, proving a poison, so we may have the spermatic, whose function is that of procreation and fecundation, performing its duty abnormally and imperfectly. Occasional instances are said to have occurred in Scotland thirty or forty years ago, and are even met with now, where the eldest son or daughter of perfectly healthy parents was impulsive, maniacal or idiotic, while all the rest of the family were quite healthy. The only cause, a good-enough one, which could be found was, the wide-spread practice of the bridegroom, and in the lower classes of the bride too, becoming quite intoxicated on the marriage evening, and conception following under such circumstances.

Atavism, again, may hold in mental as well as in bodily states. The grandchild of an unstable grandfather or grandmother, and to whom the resemblance is striking, is often found to inherit the cerebral with the physical organization; and were these matters oftener and more keenly investigated hereditary influence would be found to prevail in almost all cases of impulsive mania, especially where other causes appear defective.

With a mind predisposed to this form of disease, no influence has a greater power in setting the disordered instincts, appetites, and passions in action than example, or the law of sympathy. Cases of this kind occur in the gregarious lower animals. The monkey and parrot—proverbially imitative animals—especially when tamed, have often killed themselves in their imitative acts. In man we may mention the spread of suicide by imitation as a well-marked illustration. Assuming thus an epidemic form, it, to a certain extent, resembles hysterical affections in a ward of young females, but of course is attended by more serious consequences. In both ancient and modern times, instances of this epidemic perverted instinct have occurred. In the time of the Ptolemies, a Stoic philosopher preached so earnestly and eloquently contempt of life and the blessings of death, that suicide became frequent. The ladies of Miletus committed suicide in great numbers, because their husbands and lovers were detained at the wars. At one time there was an epidemic of drowning amongst the women of Lyons, nor could any cause be assigned for this singular tendency; it was checked by the order that all who drowned themselves should be publicly exposed in the market-place. At Miletus the mania was stopped by a similar device; the ladies chiefly hung themselves, and the magistrates ordered that in every future case the body should be dragged through the town by the rope employed for the purpose, and naked. An ancient historian of Marseilles records that the girls of that city got at one time the habit of killing themselves when their lovers were inconstant. Voluntary mutilation amongst soldiers is sometimes attributable to morbid impulse. Amongst the insane, this sympathetic impulsive tendency is common, and requires careful attention and treatment. If one patient escapes, it rarely happens but that several others are seized with the same desire. Suicides by the same means occur one after the other, and homicide is apt to have a similar tendency. After a case of the latter, I have heard several patients use threats against the special object of their suspicion and detestation, vowing that should a similar opportunity present itself, they would be impelled in the same course; and there is little reason to doubt their words. The influence of notoriety, too, on minds predisposed to morbid impulse is well known. Persons have committed murder for no other reason than that they might occupy a similar position with the felon whose doom they had just witnessed.

Cases of impulsive homicide, suicide, and rape, detailed in all their horrible minuteness by the sanguine correspondents of the daily prints, are a more frequent exciting cause to similar acts in others than is generally believed. The numerous instances of wholesale murders and suicides which have occurred of late, whether by means of secret poisoning or open violence, without doubt owe many of their victims to such mental excitants. The case of Palmer was followed by that of Dove; but the former criminal was not impulsive, the latter was on the borders, if not something more, of insanity; in fact, in a condition where morbid impulses occur with readiness. To a minor extent also the following incentives develop the tendency: the able defence of an advocate, the merciful interference of the Crown in pardoning murderers; the uncertainty of science in detecting traces of crime, as in several recent cases, and in that of the apothecary whose lawyer defended him by stating that the victim did not die of the arsenic administered by the accused, but from the vapour of the arsenical paper which bedecked his chamber walls. All these, in a mind predisposed, may hurry the person into acts of morbid impulse. This is a cogent reason why, in our asylums, all public prints are carefully scrutinized by the medical officers, and such portions excised, or the entire paper withdrawn from circulation in the institution, where morbidly suggestive or exciting matter is narrated. To do this apparently simple office well, however, requires a more extensive acquaintance with the fundamental principles of medical psychology than at first sight appears, as any one who has had an opporunity of seeing an inexperienced or ignorant person at the duty can attest. Such almost invariably cut out too much.

The sight of a suitable instrument often leads to acts of impulsive homicide and suicide; the sharp, glistening blade, and the loaded fire-arms frequently prove irresistible, and the impulsive maniac buries the one or sends the contents of the other into the living flesh of himself or others. This will explain, in a measure, why it is that persons generally commit suicide with the weapons of their trade; the sight of the ready means has been too much for them. The authorities, for this reason, have often been compelled to debar the public from ascending high towers, steeples, and monuments. A man hung himself on the threshold of one of the doors of the corridor, at the Hôtel des Invalides, in Paris. During the succeeding fortnight five invalids hung themselves on the same crossbar, and the governor was obliged to shut up the passage. For this reason it has been stated that an asylum should be so situated as to prevent the inmates from easily gaining access to, or even seeing a river, or other body of water; but this is doubtful, since the enlivening nature of a landscape so adorned would go far to compensate for the danger incurred. As an example of homicidal impulse resulting from the sight of a weapon, the following experience of the insane may be cited. A religious monomaniac had for many years an antipathy to a fellow-patient who assisted in his gallery, imagining that he practised animal magnetism, and various other "tortures of his soul" on him. He avoided him as much as possible, but he never evinced any homicidal tendency, at least so as to attract attention. So far from being suspected of such a tendency, he was, indeed, trusted with many weapons, such as cricket-bats, bow and arrows, &c., which might have been used with deadly effect on his victim had he chosen, for he was often within easy access. One rainy winter evening, however, he startled the gallery by a sudden and desperate onslaught on his victim, resulting in the death of the latter. Seeing the object of his antipathy reclining easily on a sofa and sleeping, and espying a ready and rare weapon at hand, he advanced stealthily towards him, so as to approach the sleeping person from behind, then wielding the weapon on the devoted man's head, so conveniently situated, he caused compound comminuted fracture of a fatal nature. After the act, he declared that the murdered man had ten thousand sentences of death passed upon him by the Bible, and that he was not dead, but that there existed a duplicate of him still. There was no reason to believe that his feelings with regard to the victim were different on that night from what they had been for years before, and he stated so, confessing that it was the sight of the weapon and the tempting posture of his neighbour that overcame him. Again, it is quite possible for rape to be the result of impulsive insanity; stimulated by the sight of a suitable object under favourable circumstances, an unbalanced mind may be irresistibly impelled to the gratification of the instinctive appetite. Unfortunately, most of the criminal cases of rape do not happen under such extenuating circumstances. In an asylum, the sudden introduction of strangers into the society of an inmate occasionally leads to a violent outburst of homicidal impulse, and the patient attacks the unoffending visitors with the readiest means at hand.

Another group of causes is the *blood sympathies;* the blood being the material by which all acts of supply and nutrition are accomplished, it follows that if the blood be changed in its composition, it developes violent and irregular action of the nervous system. Sometimes the want of proper nutritive materials in the food may cause it. The former is exemplified in fevers, during which the patient sometimes leaps out of a window from panphobia, or under the influence of certain drugs; the latter in the case of famishing crews cast adrift in rafts and in boats; for the starvation here as elsewhere is prone to produce impulsive acts, as homicide, suicide, insane cannibalism, &c., all the result of deranged nervous action from impoverished blood. One of the most illustrative cases on record is the following by Prof. Laycock—"A young man under my care was characterized by exalted desire for bodily activity, and an irresistible propensity to tear grass or any green thing from the ground, and eat it. For some time the symptom was not rightly interpreted, but at last it became obvious that it was a medicinal corporeal desire for a diet of fresh vegetables. The patient was therefore allowed an almost unlimited supply of raw culinary vegetables, as carrots, turnips, beet-root, cabbages, lettuce, celery, &c., which he devoured greedily in large quantities, and very soon recovered, after a prolonged attack of furious mania, during which he tore up many suits of clothes, broke hundreds of squares of glass, often assaulted his attendant and the other patients, and was altogether the most mischievous, destructive, and uncontrollable patient ever treated."* Hunger in the lower animals prompts them to the most desperate actions; and the mood of a man before dinner is proverbial. Great political changes are often or always coincident with a scarcity of provisions. In man, when there is any tendency to local disease, hunger undoubtedly acts in many cases as the exciting cause of impulsive homicide. It is thus that a liberal dietary is a most essential adjuvant in the treatment of mental disease; indeed, in not a few a full allowance of nourishing food does more good than any other individual remedy.

Diseases of the viscera, as of the liver, heart, lungs, and kidneys, may assist in the production of morbid impulse. A hypochondriacal patient may remain all his life with no further mental disorder than groundless anxiety about his health; but other stages may supervene, and by perversion of instinct he may become suicidal. He may, on the other hand, suspect that his ailments are produced by poison, galvanism, &c., and may connect some individual with this notion, and the impulse may then become homicidal. Morbid functional sympathies arising from the viscera all act by quickly altering the composition of the blood. Renal affections and cerebral disease are closely allied, and may have more connexion with the impulsive form of insanity than is generally suspected. Diseases or alterations of the intestinal canal and liver, more especially the former, may produce the affection. Suicidal impulse and erotomania have been cured by the action of a purge. Professor Laycock mentions the following case. "A most excellent friend of ours, a man of the highest moral and intellectual culture, was seized with an impulse (at church of all places) to commit an unnatural crime. Nothing could be more abhorrent to his nature, and happily for him his reason told him that such an impulse could only arise in a mind diseased. He therefore fled to us for refuge, for he knew well that if the cerebral disorder attained to such a height as at once to strengthen the foul impulse and enfeeble his will, he must fall a victim to it. The source of the morbid condition was traced to ascarides; with their destruction the horrid fiend vanished." Diseases of the arterial system, as aneurism, have caused suicidal impulse. The following case, mentioned by Prof. Miller in his lectures, may be taken as an example. A man in the Royal Infirmary, Edinburgh, suffered from iliac aneurism. An opera-

---

* *Psychol. Journal.* vol. vii., 168. p.

tion was delayed till his system—by no means a good one—might improve, but, maddened by the gnawing pain, he one day rose out of his bed and procured a corkscrew, which he thrust into the middle of the pulsating tumour. Diseases of the skin, probably from their interfering with the functions of that tissue, occasion at times a like tendency. It may follow also on tuberculosis, inflammation of the brain, or in febrile diseases involving the encephalon primarily or secondarily. We see a fever patient spring from his couch and commit homicide or suicide, or leap out of a window in a state of panphobia, as before-mentioned. The pains of parturition may cause a mild and gentle mother to lose all command over her actions, placing her for the time being in a state of impulsive mania. In certain states of cholera the same condition is asserted occasionally to present itself. Some metallic poisons, as lead, now and then give rise to impulsive insanity. In the lower animals, the instinct for self-preservation becomes perverted and morbid under certain diseases. I have seen our domestic cattle, under a disease which occasionally occurs in spring, shortly after grazing has commenced, and which is popularly known by the name of "dry fern," dash their heads violently against stone walls, posts, or trees, breaking off the bony processes of the horns and fracturing their skulls. In one instance, an animal, with its forehead quite bared of skin and its horns broken, rushed full tilt at a heap of quick lime, and buried its head therein, without manifesting additional uneasiness. In this disease the psalterium or manyplies (third stomach) has its septa so packed with hardened food, that in well marked instances, such as the above, it resembles a cannon-ball. The gastro-intestinal mucous membrane is also much inflamed, and the membranes of the brain secondarily affected.

Injuries to the brain from shocks, falls, or blows, although no inflammation or bad results follow immediately, yet it is no uncommon thing for an individual after such a lesion to have one or more of the appetites or instincts depraved or perverted. The character of the person is altered, and he is no longer the man he was before. Coup de soleil, epilepsy, and exposure to cold are stated likewise to favour impulsive actions. The influence of the weather on a community of the insane is very marked, certain impulses being brought out or aggravated by changes of temperature, the humidity and "closeness" of the atmosphere, &c. Some authors, aver, as an example of the production of impulsive insanity by certain climates, that England with its foggy one gives birth to many more suicides ; but this is not very clear; for in China, a country which is not foggy, suicide occurs on the slightest grounds. Excessive heat and cold, to a people unaccustomed to either, favours the production of certain forms of morbid impulse; the effect of the former is illustrated by the French soldiers before they were acclimatized in Spain, of the latter by the same volatile nation during the retreat of the *grande armée* from Moscow.

The influence of the sexual organs on the nervous system is well shown in that protean disease, hysteria, and scarcely less important is their bearing on this subject. As this field is a very extensive one, I shall content myself by quoting a few illustrative cases. A young woman, of industrious habits and good character, experienced an intense homicidal impulse every menstrual period. Another, whose catamenia were suppressed, attempted to strangle her neighbour, with whom she lived very happily. In almost every case of monomaniacal cunning or kleptomania among females we find some disorder of menstruation, or the connexion between the two is otherwise indicated, the former occurring most frequently in the hysterical,the latter in the parturient. Nymphomania and satyriasis depend almost entirely on the dynamical conditions of the sexual organs. Parturition, as before mentioned, is apt to upset the moral control, and frantic deeds attest the absence of the governing and restraining power. The mother, bloodless and weak, springs from her bed and madly severs head and body of her infant, and perhaps attempts her own life.

Old age, with its failing corporeal and mental conditions, becomes occasionally a cause of morbid impulse. In addition to the previous notice of this cause, the following case, recorded by Professor Laycock, may be mentioned. An old gentleman, who had all along led a virtuous life, became suddenly addicted to the most curious practices. He was observed to frequent brothels, and it was ascertained that he satiated his insane tendencies, not by sexual intercourse, but by irritating the female genitals and rectum with his fingers.

Too intense thought on religious subjects, it is stated, in the monomaniacal, is the moral cause which, next to distressed circumstances and grief, has produced the greatest number of suicides. The religious monomaniac, becomes impulsive after reading the Bible, imagining that he is ordered to slay this one and the other to aid their entrance into bliss, or, as an evidence of faith, slaughters his children or himself. He may hear voices commanding him to slay this person or that, or to burn the works of evil men. In asylums a variety of this is common—viz., the melancholiacs, who imagine that they are ordered to give up eating by angels, or that it is not right for them, so unworthy creatures, to eat like other men.

That too rigid and prolonged seclusion from society has some influence in producing morbid impulse, the following case will illustrate.* A young woman, the daughter of a lighthouse-keeper, and brought up in the secluded spot of her father's calling, married a lighthouse-keeper; thus passing most of her existence away from the society of all but two or three persons. She came to Edinburgh to see the masonic demonstration of 1858, when the multiplicity of sights, confusion of people and things in the city, so completely upset her mind, that she became an impulsive maniac, with intense desire for sexual intercourse, uttering the most obscene language imaginable, and soliciting the medical attendant. Kept in quietness for some days, with careful attention, she completely recovered her former amiable and chaste character. Solitary confinement, it is well known, increases the ferocity of the lower animals, as well as of aggressive lunatics. A preternatural sensibility to nervine excitants is also acquired by men of science who unremittingly pursue their severe labour without due intercourse with their fellow-men. Old-maid recluses, too, have sometimes remarkable propensities, such as accumulation, and a tendency to view morbidly questions of social and religious import.

The influence of a good or bad education is one of the most important points to be considered under the head of causes. A right and systematic principle of education, and an acquisition of that practical common sense which is so necessary for stability, if generally carried out, would lessen the number of cases of morbid impulse. It is the want of this useful mental and corporeal training which makes many, even highly cultivated persons, so prone to nervous disorders. When the mental culture is stimulated too much in childhood, cerebral disease is imminent; per haps not manifesting itself so much at the time as in after-life. Unzer explains that by deep and intense thought the body wastes, the blood is determined to the head, the extremities be come cold, the blood is altered in composition, and a paræsthetic condition of the nervous system results, while the viscera perform their functions imperfectly. "Hence it follows that deep studies and scientific pursuits are not the most natural objects of man, but opposed to his health and well-being. Thus it is that those learned men who cultivate the abstract sciences are generally feeble, meagre, sensitive, splenetic, hypochondrical, and fanciful, and have impaired digestion. On the contrary, the strongest and healthiest men, with good digestion, are little given to study the abstract sciences and little capable of comprehending them." Who amongst us cannot point out living examples

* Narrated by Dr. Littlejohn, in his *Lecture on Med. Jurisprudence.*

of the former statements—even in a marked form. Men of feeble mould often waste their energies in a year or two, and then spend much of their subsequent lives vainly, in search for that which they never truly possessed—viz., good health. University work has been blamed as an occasional cause of morbid impulse and insanity; but it is probable that in most cases the ordinary business of the world, sooner or later, would have upset the faculties of such constitutions as suffer from it. Curiously enough, many of those students who have impaired their mental equability by study have been those who have drunk, smoked to excess, or indulged in other vicious practices, often clearly pointing out that the original tone of the mind was at fault. We say, then, that with a tolerable constitution severe and continued study, alternating with proper physical exercise and society, is by no means favourable to the production of any species of insanity. Dr. Winslow* observes that "Blumenbach, the distinguished physician and naturalist, states, that for the long period (exceeding half a century) he was associated with the most celebrated European universities, he did not witness a solitary example of any youth falling a victim to his ardour in pursuit of intellectual distinction; and Eichhorn, one of the most voluminous writers of the day, the eminent philologist and historian, is said boldly to affirm, 'that no one ever died of hard study.'"

In the present day, the light literature everywhere so eagerly devoured, the tragic dictations of our newspapers and periodicals, the wild thirst for the play or the ball-room, the stimulants—generally so unnecessary—used for drink by all classes, and many other common pleasures and vices—it is these, and not the profession or pursuit, that saps the mind into a state suitable for morbid impulse. Again, with regard to the education of our women, well has it been said, "The vices of the education adopted by our young ladies, the preference given to acquirements purely ornamental, the reading of romances, which gives the intellect a precocious activity and premature desires, together with ideas of an imaginary excellence which can never be realized; the frequenting of plays and society, the abuse of music, and want of occupation, are causes rendering insanity very frequent among our women." Nymphomania, no uncommon disease in institutions for young ladies, may surely be in part attributed to the above and to that rigid seclusion of the young and susceptible female which prevents a salutary and virtuous intermingling in the society of the other sex, and to

* Page 693, *Obscure Diseases of the Brain, &c.*

the want of due appreciation of her duties—morally and physically—in this life.

The abuse of alcoholic stimulants is another cause of morbid impulse, either by hereditary influence, as we have previously seen, or in the individual singly. The drunken parent, the wretched slave of a meaningless and degraded passion, cannot but procreate beings with some moral blight, prone to crimes and impulses. This is seen in its most deplorable condition in the lower classes, where of such children the girls are the more to be pitied, since they—ignorant and impulsive—are readily seduced by abandoned men. In the better classes, examples are by no means rare where certain members of a family, descended from a drunken parent or parents, are guilty of acts evidently of insane impulse, as extreme licentiousness, cruelty, murder, or suicide, a lasting witness of their parent's inebriation. The vast number of crimes which occur under the influence of intoxication makes further comment on its effects upon the individual unnecessary.

Remarkable cases occur not unfrequently in which the person suddenly rises out of sleep and commits homicide and suicide. In such we must regard the peculiar mental state during sleep as the cause of the impulse. Suddenly awakening from sleep, and perhaps from frightening dreams, the person believes he sees a robber attacking him, and seizes a weapon and slays him in a moment; on recovering his usual state of mind he finds his own wife or friend bleeding in bed beside him. In his dream, an attack by robbers may have formed the theme, he suddenly awoke, the form of his sleeping companion caught his eye, and at once the ideal of his dream became personified and the tragedy accomplished—alike the thought and action of an insane person. The case of the pedlar already mentioned is a good instance. Professor Laycock* relates the case of a harlequin, who was accustomed to leap through a window when performing on the stage; he fell asleep in a railway carriage close by the open window, and suddenly awakening sprang out of the carriage. Fortunately he did not sustain serious injury by the impulsive act. In this instance, the sight of the open window and his previous employment at once suggested the leap, which was not restrained by the thought of danger or of his present position. The same high authority mentions the case of a patient of his in the Royal Infirmary, Edinburgh, who was afflicted with aortic insufficiency and dropsy, and who conceived a great desire to terminate his existence just before or immediately after

---

* *Clinique of Mental Diseases.*

sleep, and yet, so far from loving death, he had a great notion of living. The well-known cases of Bernard Schedmaizig, who murdered his wife in bed;* of a woman who rose from sleep and cut the throat of her child; of a lady who rose in the middle of the night and drowned herself in a cistern of water;†—all come under this head. The mental faculties of the person in this condition are in the state found in dreaming, while in addition, impressions from the sight of surrounding objects affect him, and may give rise to impulsive acts, particularly in persons of such mental constitutions as we have frequently indicated. We see in the bewildered gaze and hurried manner of a newly awakened sleeper a person partially insane.‡ It is curious and interesting in asylum life to notice the bearing of certain patients when thus suddenly roused. One old man—a case of chronic dementia, and who is for the most part mute, only uttering a grunt now and then if annoyed or terrified—springs, when awakened, in the most agile manner from his bed, and talking loudly, rushes for a considerable distance without motive or object. A feeling of alarm is the most common mental manifestation, which in certain cases of monomania and chronic mania is apt to assume an aggressive form. Attendants on the insane who sleep with them in dormitories are often assaulted during the night by patients who rise from bed with a homicidal inclination.

Other causes of morbid impulse are stated to be, want of steady purpose, indiscretion, love of excitement, frivolity, self-esteem; as in the case of a person who imagined himself an actor, and who was painfully mortified on finding no one else did, and seizing a pistol blew out his brains. Also, the malignant passions, pride, fear, fright, political commotions, military and despotic governments, where suicides are frequent; want of sleep, anxiety, ambition, reverses of fortune, domestic troubles—a very common origin of suicidal impulse; shame and mortification, as in the case of a young lady who was violated—she drowned herself; excessive modesty, as in some women who become insane on the night of their marriage. Congenitally defective states of the brain, as idiocy and imbecility, are the causes of many acts of insane impulse. Civilization is held to favour the production of insanity, especially insanity due to moral causes. The epidemic and impulsive insanity of an epoch bears impress of the dominant ideas and events of the age; and the

---

* Pagau, *Med. Jurisp of Insanity.* † Winslow, *Obscure Diseases, &c.*

‡ Vide *Journal of Psychol. Med.*, 1851, *On Sleep, Dreaming, and Insanity*, by Prof. Laycock.

increase of insanity is in relation to the development of the intellectual faculties, of the passions, of industry, of riches, and of misery. In proportion as men are exposed to the influences which excite the passions, so these are apt to become strong, and at last lead the individual into undoubted acts of insanity.*

---

### Art. IX.—THE SICKNESS AND MORTALITY OF THE FEDERAL VOLUNTEERS.

* Vide *Journal of Psychological Medicine*, April, 1854, *On Some of the Latent Causes of Insanity.*

